# Ellenberg Indicator Values Disclose Complex Environmental Filtering Processes in Plant Communities along an Elevational Gradient

**DOI:** 10.3390/biology12020161

**Published:** 2023-01-19

**Authors:** Letizia Di Biase, Noelline Tsafack, Loretta Pace, Simone Fattorini

**Affiliations:** 1 Department of Life, Health and Environmental Sciences, University of L’Aquila, Via Vetoio, 67100 L’Aquila, Italy; 2 cE3c–Centre for Ecology, Evolution and Environmental Changes, Azorean Biodiversity Group, Faculty of Agricultural Sciences and Environment, CHANGE-Global Change and Sustainability Institute, University of the Azores, Rua Capitão João d’Ávila, Pico da Urze, 9700-042 Angra do Heroísmo, Portugal

**Keywords:** Apennines, community ecology, community-weighted mean, CWM regression, fourth-corner analysis, Italy, Mediterranean, mountains, multi-level modelling, niche

## Abstract

**Simple Summary:**

Plant species of a regional flora have different ecological preferences, leading to the presence of different assemblages along environmental gradients. Botanists elaborated score systems to express species preferences for environmental factors, such as temperature, light, soil moisture, etc. The most popular system is that of the ‘Ellenberg indicator values’ (EIVs). EIVs have been largely applied to use plant species as indicators of environmental characteristics. In this research, we adopted a different perspective, and used EIVs to study how species are filtered by variations in ecological conditions along an elevational gradient. We used the flora of a small mountain in Central Italy as our case study. We found that heat-loving species are progressively replaced by cold-adapted ones at increasing elevations. Sunlight-adapted species prevail at low and high elevations (where open habitats occur), whereas in the middle of the gradient (occupied by the beech forest) shade-loving species predominate. Variation for moisture and soil nutrient preferences followed a similar pattern since humus abundance makes forest soils moister and richer in nutrients. Preferences for pH and continentality did not follow any clear pattern, since these factors are subject to more local variations. These results highlight the possible use of EIVs to study how plant communities respond to environmental gradients.

**Abstract:**

Ellenberg indicator values (EIVs) express plant preferences for temperature, light, continentality, soil moisture, pH, and soil nutrients, and have been largely used to deduce environmental characteristics from plant communities. However, EIVs might also be used to investigate the importance of filtering mechanisms in shaping plant communities according to species ecological preferences, a so far overlooked use of EIVs. In this paper, we investigated how community-weighted means (CWM), calculated with EIVs, varied along an elevational gradient in a small mountain in Central Italy. We also tested if species abundances varied according to their ecological preferences. We found that the prevalence of thermophilous species declines with elevation, being progressively replaced by cold-adapted species. Heliophilous species prevail at low and high elevations (characterized by the presence of open habitats), whereas in the middle of the gradient (occupied by the beech forest), sciophilous species predominate. Variations for moisture and soil nutrient preferences followed a similar pattern, probably because of the high moisture and nutrient levels of forest soils with a lot of humus. No distinct pattern was detected for EIVs for pH and continentality since these factors are subject to more local variations. These results highlight the possible role of EIVs to investigate how environmental gradients shape plant communities.

## 1. Introduction

Plant species distribution and abundance are constrained by several abiotic factors, mainly represented by climatic conditions (such as light, temperature, and precipitation) and soil characteristics (such as nutrient contents, pH, and chemical composition) [1,2,3]. Species responses to these variables define their ecological tolerance (the range of conditions in which the species can survive) and optimum (the value that is optimal for species’ existence, development, growth, and reproduction) [3,4,5].

While tolerances and optima define the fundamental niche of a species, the realized niche includes the effects due to the presence of other organisms (such as competitors and facilitators) [1,3,6,7,8,9,10,11,12]. Thus, the observed preferences shown by species in communities cannot necessarily reflect their ideal optima but express their realized ecological optima.

To denote their ecological preferences, plant species can be associated with a particular gradient of abiotic conditions and can receive a value indicating the position at which each, on average, reaches a peak of abundance along this gradient [13], that is its realized optimum [14,15]. Using this approach, Ellenberg [16,17,18] proposed a system of “indicator values” for the Central European flora (updated by Ellenberg et al. [19,20,21]), in which species preferences (realized optima) to edaphic and climatic parameters are evaluated in comparison with other species using ordinal scales. Specifically, Ellenberg indicator values (EIVs) consider species preferences for the following environmental parameters: light availability, temperature, climatic continentality, soil moisture, reaction (soil or water acidity/pH), nitrogen (in fact, soil fertility or productivity, and not mineral nitrogen), and salinity. Since EIVs are based on field observations of species distributions, and species behavior may differ even widely from one region to another, calibrations have been introduced for different floras [22,23,24,25,26,27,28,29,30,31,32,33].

EIVs are the most commonly used score system to express plant ecological preferences and are largely used for bioindication, that is, to drive conclusions about the environment from the species composition of a given community (e.g., [34,35,36,37,38,39]). With this approach, EIVs are used as surrogates for measured environmental variables [40,41,42,43,44]. By contrast, EIVs have been relatively little used as aids to the interpretation of spatial and temporal vegetation patterns [45,46,47,48].

Some research has been conducted on the relationships between EIVs and variations in environmental parameters at the community level. For example, Schaffers and Sýkora [49] correlated EIVs with field measurements, finding that the EIVs for moisture correlated positively with the average lowest moisture contents in summer, annual average groundwater level, and average spring level; EIVs for nitrogen were only weakly correlated with nitrogen mineralization and available mineral nitrogen, but were strongly correlated with biomass production; EIVs for pH were not correlated with soil pH, but showed a strong correlation with the total amount of calcium. Wamelink et al. [50] found a positive relationship between EIVs for pH and soil pH, and a negative relationship between EIVs for moisture and mean spring groundwater level; however, the regression parameters were influenced by the type of vegetation. It has also been observed that the EIVs for pH may be a good predictor of species richness for Central European vegetation, with the shape of relationship being however positive, negative, unimodal, or even absent, according to the vegetation type [51]. In a study conducted by Sørensen and Tybirk [52], the EIVs indicated an increase in nitrogen availability and a decrease in acidity and light availability through the secondary succession from a heath to an oak forest. Lososová et al. [53] investigated how EIVs in arable lands responded to variations in elevation, growing season, and long-term changes (from small fields to vast tracts of arable land with intensive management), finding that the EIVs for light, temperature, continentality, pH, and nutrients decreased with elevation, while the EIVs for moisture increased. By contrast, all these EIVs increased with the season except for the EIVs for pH. Finally, all the EIVs increased with long-term changes, except those for temperature and continentality. Fraaije et al. [54] found that patterns in germination, seedling survival, and seedling growth along a riparian gradient varied among plants with different EIVs for moisture. Marcenò and Guarino [48] found that in Mediterranean evergreen woods, precipitation positively correlated with the EIVs for continentality (albeit poorly), moisture, and nitrogen, and negatively with the EIVs for light and temperature; temperature was correlated negatively with the EIVs for continentality (albeit poorly), moisture, and nitrogen, and positively with the EIVs for light and temperature (correlations with EIVs for pH were non-significant). Chytrý et al. [33] found that, in the Czech flora, the EIVs for light were negatively correlated with the percentage of the tree layer cover, the EIVs for temperature were positively correlated with the mean July temperatures, the EIVs for moisture were positively correlated with precipitation, the EIVs for pH were positively correlated with pH, and the EIVs for nutrients were negatively correlated with the carbon: nitrogen ratio. Very recently, Kutbay and Surmen [55] investigated how EIVs varied along a sea–inland gradient in coastal dune vegetation in the Central Black Sea Region of Turkey, showing that the EIVs for salinity and pH decreased along the gradient, while nutrient content EIVs increased. These studies indicate that the EIVs at community level reflect environmental conditions that vary along gradients, and thus that EIVs might be used to investigate how community structure is influenced by plant responses to environmental gradients. Quite surprisingly, however, this approach has been so far substantially unexplored.

In this paper, we investigated how plant communities vary along an elevational gradient according to their ecological preferences defined by the EIVs. In mountain areas, many environmental characteristics (from climate conditions to soil properties) show large variations within a small geographical area, making elevational gradients ideal to investigate hypotheses about the influence of environmental variables on biodiversity patterns and ecological processes [56,57,58,59,60,61]. The patterns of plant community structure are typically discussed via various filtering mechanisms, in which environmental conditions sort the species that fulfill local niche requirements [6,62,63]. Assuming that communities change with elevation as a result of the filtering effects of environmental factors on common species pools, EIVs can therefore be profitably used to investigate how elevation filters species according to their preferences for a variety of environmental gradients.

Using this approach, we tested the following hypotheses:

(1) The EIVs for temperature should decrease with increasing elevation, following the decrease of temperature with increasing elevation (for the temperate zone summer, there is a drop of about 0.6 °C for every 100 m above sea level [56]). Thus, thermophilous (warm-adapted) species (i.e., plants with high EIVs for temperature), which should dominate low-elevation communities, are expected to be replaced by species with progressively lower EIVs (from mesophilous species, adapted to intermediate conditions, to cryophilous species, i.e., cold-adapted species).
(2) The EIVs for light should increase with elevation, because light intensity (solar radiation) tends to increase with elevation. Lower air density and particulate matter at higher altitudes translate into greater solar radiation [56]. Additionally, with increasing elevation, vegetation becomes sparse and reduced to few herbaceous species [59]. This means that the shadow provided by trees is progressively reduced and eventually lacking. Therefore, sciophilous species (i.e., shade-loving plants) are expected to be replaced by progressively more heliophilous species (i.e., species adapted to higher levels of direct sunlight).
(3) The EIVs for moisture should increase with elevation, because, at least in the temperate zone, precipitation tends to increase with elevation, which should translate into a higher soil moisture [56].
(4) The EIVs for nutrients should decrease with elevation because soils become less fertile at higher elevations. With an increasing elevation, soil decomposition becomes slower, and since higher slopes tend to become progressively steeper, rain and melting snow carry away more and more soil, making soil thinner and less fertile [56,59]. Thus, species that need a high concentration of soil nutrients are expected to be progressively replaced by those able to survive in soils with low levels of phosphorous, nitrogen, and organic matter.
(5) The EIVs for soil reaction (pH) should increase with elevation because of decreasing values of soil pH. Soil pH tends to decrease with elevation due to the slow decomposition of organic matter (which releases acids) and higher precipitation, which increases the leaching of basic cations [64,65,66,67,68].
(6) The EIVs for continentality are not expected to show any distinct variation with elevation, since they tend to not exhibit recognizable patterns of spatial variation and dependence on environmental variables [48,69,70,71]. The concept of continentality integrates thermic and hygric gradients and may reflect geographical proximity to the ocean, as well latitudinal and altitudinal gradients, since the ecological importance of temperature increases toward higher latitudes and altitudes, while the importance of humidity increases towards lower latitudes and altitudes [69]. However, the EIVs for continentality rarely provide meaningful results and were used less frequently than any other EIVs [69]. In particular, studies using the EIVs on a large scale typically did not consider continentality, and its use in small-scale studies only provided barely interpretable results [69,70,71]. Given the very small scale of our study, we do not expect any meaningful variation of continentality values with elevation.

## 2. Materials and Methods

### 2.1. Study Area and Data Collection

We used data from 16 relevés (sites) taken from a phytosociological study [72] conducted in a natural reserve (“Monte Genzana e Alto Gizio”, 3160 hectares) in Central Italy (41°56′53.37″ N–13°53′14.91″ E). The reserve is located in the inner part of the Central Apennines and has an elevational range spanning from 530 m to 2170 m. From a geological point of view, the area is mainly occupied by dolomite and limestone [72,73]. In general, soils present in the study area have a mollic epipedon, very low available water capacity, medium texture (loam, sandy loam, or loamy sand soils), and very high organic matter provided by forest vegetation (e.g., beech forest); however, well drained, rocky soils with medium texture (from silt to sandy loam soils) are found on carbonate reliefs over 1600 m elevation [73]. The area has a temperate-continental climate, with temperature declining regularly with elevation by about 0.6 °C every 100 m (personal observations in autumn 2022). Because of the remarkable extent of its elevational range, the area encompasses forms of vegetation from all vegetational belts that can be found on the Apennines: thermophilous woods in the lowlands and hilly lands, dominated by downy oak (*Quercus pubescens* Willd.) and European hop-hornbeam (*Ostrya carpinifolia* Scop.); beech (*Fagus sylvatica* L.) forests (from 1000 to 1800 m); subalpine shrublands; and high-montane grasslands. A brief description of the plant community of each relevé used in this study is given in Table 1. Further details on the vegetation of the study area can be found in Pirone [72] and Di Biase et al. [74]. Taxonomy follows Pignatti et al. [75].

To express species abundances, we converted original scores based on the seven-grade Braun–Blanquet scale [77] to percentage cover as follows [78,79,80]: r = 1%, + = 2%, 1 = 3%, 2 = 13%, 3 = 38%, 4 = 63%, and 5 = 88% (however, no species was ranked as r in the original phytosociological study). Because in the original phytosociological study cover data were recorded separately for different strata, we constructed and analyzed two separate matrices: one including only the shrubby-herbaceous stratum, as already conducted in a previous paper, in which only presence/absences were used ([74], with corrections), and the other also including the arboreal stratum. When a species was present in more than one stratum with different values of cover, we considered the maximum value.

We assigned to each species the respective EIVs following Pignatti et al. [25] and Guarino et al. [81]. We considered EIVs for the following preference gradients (extreme values are reported as an indication of the ranges as defined for the Italian flora; the ranges for the species considered in this study are given in parentheses):

L—light: 1 (species growing in sites with dense shade, up to 1% of external light; 30% of external light can be recorded for short periods) to 12 (plant growing in full sun, in sites with high irradiation, low haze climate, and presence of reflection effects) (2–11).

T—temperature: 1 (species associated with cold environments, only occurring at high elevations or with Arctic–Alpine distribution) to 12 (South Mediterranean species associated with warm places and subdesert environments) (2–9).

K—climatic continentality: 1 (oceanic species occurring as relict populations) to 9 (species mainly distributed in areas with continental climate, occurring in Italy with disjunct populations) (3–9).

F—soil moisture: 1 (species that can live only in arid places and associated with dry soils) to 12 (plants that live submerged, at least for long periods) (1–9).

R—reaction (soil or water acidity/pH): 1 (species associated with very acidic soils) to 9 (species associated with strongly alkaline substrates) (2–9).

N—nutrients: 1 (species able to survive in oligotrophic conditions, associated with soils with very low content of phosphorus, nitrates, and organic matter) to 9 (species living in environments with excessive concentrations of phosphorus and nitrogen, such as landfills) (1–9).

Salinity was excluded because it has no meaning outside coastal regions and preference for salinity is unknown for almost all the species considered in this study. For each gradient, we used the symbol X to indicate species for which the respective EIV was not available because of their broad ecological preferences (uninformative species). DD (data deficient) was used for species of unknown preference. Species cover (%), EIVs and elevation of relevés are given in Tables S1–S5.

### 2.2. Data Analysis

We conducted separate analyses for the L, T, K, F, R, and N preference gradients. Species categorized as X or DD for a certain preference gradient were excluded from the respective analyses, thus the total number of species analyzed varied according to the gradient considered.

For each preference gradient, we investigated how plant preferences at the community level varied with elevation by using community-weighted mean (CWM) values [5,82,83,84]. Since values of species’ ecological preferences are weighted toward the dominant species in the community, CWM values based on EIVs characterize the most important response of a community to a given environmental variable.

For each preference gradient, CWM was computed as:

(1) $$\mathrm{CWM}=\sum_{i=1}^{S}t_{i}p_{i}\;,$$

where *S* is the number of species in the community, *t_i_* is the EIV of the *i*th species, and *p_i_* is the relative cover of the *i*th species.

CWM values were then regressed on elevation to model how average plant species preferences change along the elevational gradient. CWM regressions have been used widely to assess which functional traits are most strongly explained by changes in environmental variables along gradients (e.g., [85,86,87,88,89,90]), and they are applied here to investigate changes in environmental preferences.

However, CWM regressions suffer from inflated type I error rates because of the lack of independence of CWM values among samples that contain the same species [91,92]. To address this lack of independence of CWM traits (in our case, ecological preferences expressed by EIVs; however, we used the word ‘trait’ for simplicity), the significance of trait–environment relationships can be assessed by randomizing the location of species abundances in the matrix [93]. This approach, known as fourth-corner analysis, reduces type I error rates and increases statistical power [91,94]. Thus, we complemented the CWM regressions with fourth-corner analyses for the evaluation of the significance of correlations.

Finally, we adopted a multi-level model approach [95,96] in which species’ ecological preferences are used as predictors of species abundance. This approach does not aim at testing whether variations of community-level trait averages (in our case, ecological preferences expressed by EIVs) along a gradient result from an environmental filter, but tests whether the relationship between species abundances and environmental characteristics depends on the preferences of the species. With this approach, we tested whether a species with a given preference for one of the ecological gradients considered by EIVs is more likely to occur in one part of the elevational gradient over another. To remove the effect that trait values are measured on species that occur in multiple sites (‘species effect’), multi-level models use traits and environmental conditions as fixed effects and species as random effects [97]. Following Laughlin et al. [96,98], we included a trait–environment interaction as a fixed effect in the model to test whether the effect of elevation on the occurrence of a species depends on its ecological preference, while allowing species abundances to vary along the elevational gradient as a random slope to control for the ‘species effect’. This approach tests whether traits (in our case, ecological preferences expressed by EIVs) affect species abundances in response to environmental conditions, while simultaneously controlling variation in species distribution along the gradient. We applied the multi-level modelling approach by fitting generalized linear mixed models (GLMMs) using a binomial error structure and a log link function to model the presence and absence of species along the elevational gradients. The so-called fixed effects included the interaction between EIVs and elevation. Random effects included a random intercept for each site to account for variation in occurrences across sites, random intercepts for each species, and random slopes for elevation to account for variation in species occurrences along the gradient.

When non-linear patterns (either hump-shaped or U-shaped) were detected, we divided the gradient into sections that could be adequately fitted by linear models. Specifically, we subdivided the overall gradient into two subgradients: 600–1200 m and 900–2000 m, because peaks for hump-shaped patterns or minimums for U-shaped patterns were at around 1000 m. The two subgradients overlapped at elevations 900–1200 m. This choice is justified by the following rationale. First, relevés at 900–1200 m consisted of three forest sites that are representative of the forest vegetation that occurred around this elevation. In the scatterplots, they clustered very closely and assigning some of them to one subgradient and the remaining to the other would be arbitrary. Second, this choice relays on biological grounds. The 900–1200 m range falls in the vegetation belt dominated by beech forests in Central Italy [72]. These forests represent a hinge between the more thermophilous vegetation of lower elevations and the open vegetation of higher elevations.

To summarize, we used: (1) CWM regressions to model variation in community EIVs along gradients; (2) fourth-corner analyses to evaluate the significance of correlations; and (3) multi-level modelling to determine which species preferences are selected along environmental gradients. All calculations were performed in R [99], adapting the code prepared by Daniel Laughlin for community trait analysis [100]. Specifically, we used the function functcomp of the R package FD [101] to calculate CWM values. Community matrices were previously standardized using the function decostand, with the method ‘total’ in the R package vegan [102]; the function lm (of stats package, which is part of R) was used for linear regression; the function fourthcorner of the R package ade4 [103] was used for the fourth-corner analyses; and the functions glmer of the R package lme4 [104] and anova (of stats package) were used for the multi-level analyses. Traits (i.e., EIVs) and elevation values were scaled prior to fit GLMMs models. We first fitted a model without interaction and then fitted a second model with interaction between EIVs and elevation. The allFit function of the R package lme4 was used to investigate the best optimizer for each model. The function anova was used to test if models with and without interaction were different (i.e., to see if adding interaction significantly improved the model). The function r2 of the R package performance [105] was used to compute conditional and marginal R^2^ values of mixed models. To fit the GLMM environment-only models, the following model was used: Binomial Presence/Absence ~ Environment + (Environment|Species) + (1|Site), with the option control = glmerControl (optimizer = “…”). To fit the GLMM trait × environment model, the following model was used: Binomial Presence/Absence ~ Trait × Environment + (Environment|Species) + (1|Site), with the option control = glmerControl (optimizer = “…”). The optimizing function “bobyqa” was used as optimizer for most of the models, but for some models we used the functions “Nelder_Mead”, “nloptwrap” or “nmkbw” to obtain convergence. For further details, the reader can inspect the code of Daniel Laughlin [100].

## 3. Results

We obtained very similar results including or excluding the arboreal stratum. Thus, in the following section we only report the results with trees. Results without trees are given in Figures S1–S6. Full numerical details of the results obtained for both datasets are given in Tables S6–S17.

**Figure 1:**
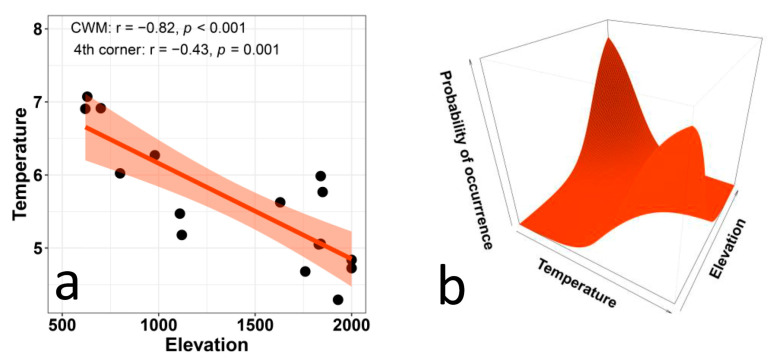
Relationship between Ellenberg indicator values for temperature and elevation in plant communities along an elevational gradient in Central Italy. The left panel (**a**) presents the CWM regression model and statistical corrections based on the fourth-corner analysis. The right panel (**b**) presents the results of the multi-level model (trait × environment interaction *p* < 0.001, marginal R^2^ = 0.06).

As expected, CWM values of temperature preferences (Figure 1a) decreased distinctly with elevation, and the fourth-corner analysis supported the statistical significance of this relationship. Results from the multi-level approach (Figure 1b) showed a strong relationship between plant preferences for temperature and elevation: species that prefer high temperatures occurred at lower elevations and species that prefer low temperatures occurred at higher elevations. The ANOVA results indicate that the trait × environment interaction significantly improves the model (*p* < 0.001), whereas the fixed effects only explain 6% of the variation.

**Figure 2:**
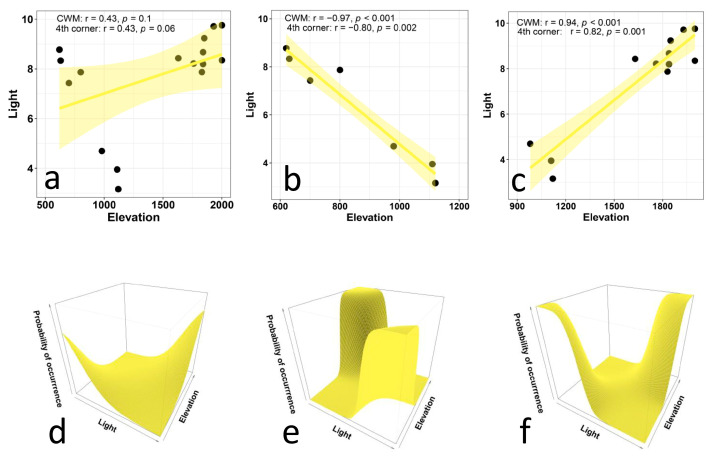
Relationship between Ellenberg indicator values for light and elevation in plant communities along an elevational gradient in Central Italy. The upper panels (**a**–**d**) present CWM regression models and their statistical corrections using fourth-corner analysis for the entire gradient (**a**), for the lower subgradient (**b**), and for the upper subgradient (**c**). The lower panels (**d**–**f**) illustrate the results of multi-level models for the entire gradient (**d**, trait × environment interaction *p* < 0.001, marginal R^2^ = 0.03), for the lower subgradient (**e**, trait × environment interaction *p* < 0.001, marginal R^2^ = 0.26), and for the upper subgradient (**f**, trait × environment interaction *p* < 0.001, marginal R^2^ = 0.32).

We detected a weakly positive (non-significant) correlation between the CWM values for light preference and elevation (Figure 2a). In fact, plant preferences for light seem to have a U-shaped pattern, because of the presence of very low values of CWMs at intermediate elevations. This partially contrasts with our hypothesis of a positive correlation. Therefore, we divided the overall gradient into two subgradients, and conducted separate analyses for each of them. These analyses clearly indicated that the CWM values for light were negatively correlated with elevation in the first subgradient (Figure 2b) and were positively correlated with elevation in the second subgradient (Figure 2c).

The results from the multi-level approach (Figure 2d–f) showed a strong relationship between plant preferences for light and elevation in both subgradients. In the first subgradient (Figure 2e), species that prefer high levels of light occurred at lower elevations, and species that prefer low levels of light occurred at middle elevations. The ANOVA results indicated that the trait × environment interaction significantly improves the model (*p* < 0.001), and the fixed effects explain about 30% of the variation. In the second subgradient (Figure 2f), species that prefer low levels of light occurred at middle elevations, and species that prefer high levels of light occurred at higher elevations. The ANOVA results indicate that the trait × environment interaction significantly improves the model (*p* < 0.001), and the fixed effects explain about 26% of the variation.

**Figure 3:**
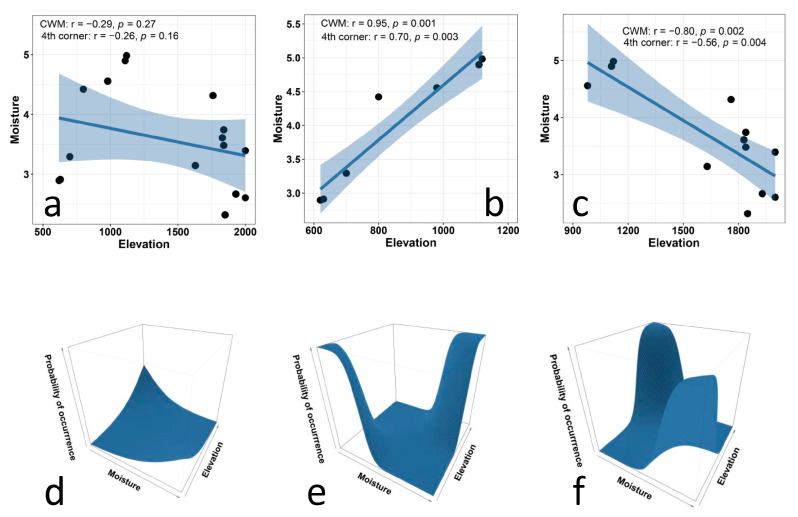
Relationship between Ellenberg indicator values for moisture and elevation in plant communities along an elevational gradient in Central Italy. The upper panels (**a**–**d**) present CWM regression models and their statistical corrections using fourth-corner analysis for the entire gradient (**a**), for the lower subgradient (**b**), and for the upper subgradient (**c**). The lower panels (**d**–**f**) illustrate the results of multi-level models for the entire gradient (**d**, trait × environment interaction *p* < 0.01, marginal R^2^ = 0.02), for the lower subgradient (**e**, trait × environment interaction *p* < 0.001, marginal R^2^ = 0.21), and for the upper subgradient (**f**, trait × environment interaction *p* < 0.001, marginal R^2^ = 0.13).

**Figure 4:**
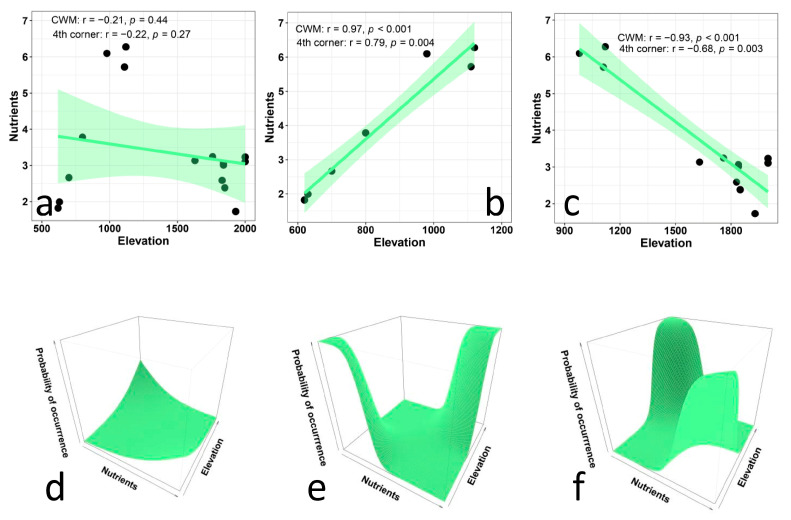
Relationship between Ellenberg indicator values for nutrients and elevation in plant communities along an elevational gradient in Central Italy. The upper panels (**a**–**d**) present CWM regression models and their statistical corrections using fourth-corner analysis for the entire gradient (**a**), for the lower subgradient (**b**), and for the upper subgradient (**c**). The lower panels (**d**–**f**) illustrate the results of multi-level models for the entire gradient (**d**, trait × environment interaction *p* < 0.01, marginal R^2^ = 0.01), for the lower subgradient (**e**, trait × environment interaction *p* < 0.001, marginal R^2^ = 0.13), and for the upper subgradient (**f**, trait × environment interaction *p* < 0.001, marginal R^2^ = 0.15).

The correlation between the CWM values for soil moisture preferences and elevation (Figure 3a), as well as that between the CWM values for nutrients and elevation (Figure 4a), are extremely low. In fact, in both cases, the CWM values show unimodal patterns, because of the preference of very high values of CWMs at intermediate elevations. These results contrast with our expectations of a positive correlation between elevation and moisture preference and a negative correlation between nutrients and elevation. Therefore, we divided the overall gradients into two subgradients, and conducted separate analyses for each of them for both moisture and nutrients.

For moisture, these analyses clearly indicated that the CWM values were positively correlated with elevation in the first subgradient (Figure 3b) and were negatively correlated with elevation in the second subgradient (Figure 3c). Results of the multi-level approach (Figure 3d–f) showed a strong relationship between plant preferences for moisture and elevation in both subgradients. In the first subgradient (Figure 3e), species that prefer low levels of moisture occurred at lower elevations, and species that prefer high levels of moisture occurred at middle elevations. The ANOVA results indicate that the trait × environment interaction significantly improves the model (*p* < 0.001), and the fixed effects explain about 20% of the variation. In the second subgradient (Figure 3f), species that prefer high levels of moisture occurred at middle elevations, and species that prefer low levels of moisture occurred at higher elevations. The ANOVA results indicate that the trait × environment interaction significantly improves the model (*p* < 0.001), and the fixed effects explain only about 13% of the variation.

For nutrients, the separately conducted analyses for the two subgradients clearly indicated that CWM values were positively correlated with elevation in the first subgradient (Figure 4b) and were negatively correlated with elevation in the second subgradient (Figure 4c). Results from the multi-level approach (Figure 4d–f) showed a strong relationship between plant preferences for nutrients and elevation in both subgradients. In the first subgradient (Figure 4e), species that prefer low levels of nutrients occurred at lower elevations, and species that prefer high levels of nutrients occurred at middle elevations. The ANOVA results indicate that the trait × environment interaction significantly improves the model (*p* < 0.001), and the fixed effects explain about 23% of the variation. In the second subgradient (Figure 4f), species that prefer high levels of nutrients occurred at middle elevations, and species that prefer low levels of nutrients occurred at higher elevations. The ANOVA results indicate that the trait × environment interaction significantly improves the model (*p* < 0.001), and the fixed effects explain only about 15% of the variation.

**Figure 5:**
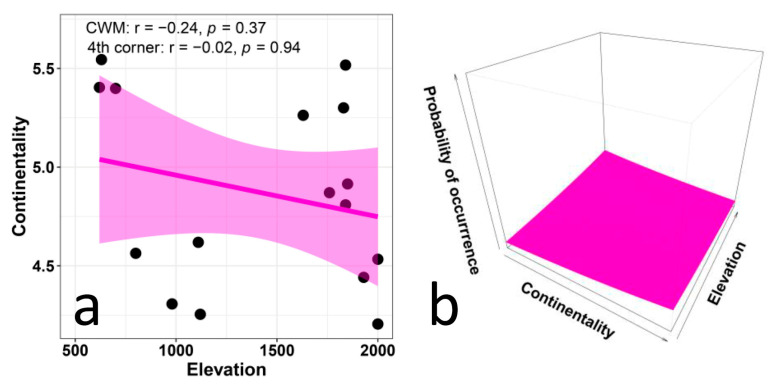
Relationship between Ellenberg indicator values for continentality and elevation in plant communities along an elevational gradient in Central Italy. The left panel (**a**) presents the CWM regression model and statistical corrections based on the fourth-corner analysis. The right panel (**b**) presents the results of the multi-level model (trait × environment interaction *p* < 0.001, marginal R^2^ = 0.001).

**Figure 6:**
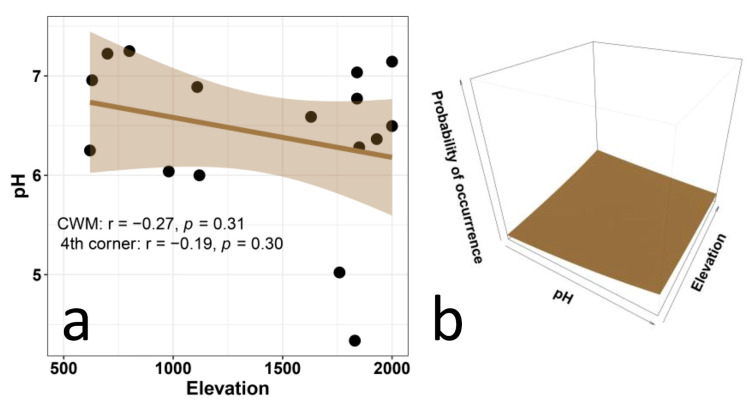
Relationship between Ellenberg indicator values for reaction (pH) and elevation in plant communities along an elevational gradient in Central Italy. The left panel (**a**) presents the CWM regression model and statistical corrections based on the fourth-corner analysis. The right panel (**b**) presents the results of the multi-level model (trait × environment interaction *p* = 0.45, marginal R^2^ = 0.003).

Elevation did not influence the CWM values of continentality (Figure 5a) and reaction (pH) (Figure 6a) in any obvious way. The results of the multi-level approach also show no significant relationship between continentality and elevation (Figure 5b) and between reaction (pH) and elevation (Figure 6b). The results for continentality conform to our hypothesis of a lack of relation, while those for pH are in contrast with our expectation of a positive correlation.

## 4. Discussion

In accordance with our hypothesis, temperature preferences, expressed by the CWM values, showed a distinctly inverse relationship with elevation, which can be explained by the thermal gradient (temperature decreases with elevation). Thus, thermophilous species (which dominate the vegetation at lower elevations) are progressively replaced by cold-adapted species. This is clearly shown by the probability of species occurrence, which shows two peaks: one at low elevation–high temperature (which is related to warm-adapted species that dominate low elevation communities) and one at high elevation–low temperature (which is related to the dominance of cold-adapted species in high elevation communities). This pattern paralleled the biogeographical patterns observed by Di Biase et al. [74], in which the proportion of species with Mediterranean distributions (which are expected to be more thermophilous) declined along the elevational gradient, whereas that of Euromontane and Mediterraneo-Montane species (which are expected to be more cold-adapted) increased with elevation.

The EIVs for light were positively corelated with elevation, but this relationship was weak and non-significant, which partially contrasts with our hypothesis of a positive correlation. Elevation is a poor correlate of light preferences under the assumption of a linear relationship because of the preponderance of sciophilous species at around 1000 m, which generates a U-shaped pattern. This can be related to the concentration of forest vegetation at intermediate elevations. As forests are shady places, it is not surprising that plants of forest vegetation are sciophilous. By contrast, the prevalence of open environments at the lower elevations (ca 600–700 m, where garigues of the *Cytiso spinescentis-Satureion montanae* alliance occurs [72]) and at the higher elevations (ca 1600–2000 m, where dry semi-natural mountain grasslands belonging to the *Festuco valesiaceae-Brometea erecti* class and open high-mountain grasslands belonging to the *Festuco-Seslerietea* class prevail [72]) explains the preponderance of heliophilous species at the two extremes of the gradient. When the overall gradient was divided into two subgradients, we found that the CWM for light decreased with an increasing elevation in the first subgradient and increased in the second one. This is reflected by the species distributions outlined by the multi-level analyses, which showed that sciophilous species predominate at middle elevations, being progressively replaced by heliophilous species at lower and higher elevations. It is important to stress that the prevalence of certain grasses at high elevations might have been emphasized by anthropogenic causes (in particular, the abandonment of pastoral activities), with some dominant species, such as *Brachypodium genuense* (DC.) Roem. et Schult and *Sesleria nitida* Ten., influencing the community composition by the competitive exclusion of subordinate species [106] beyond the filtering effects determined by variations in natural environmental conditions.

Contrary to our expectations, preferences for both moisture and nutrients did not correlate linearly with elevation, showing unimodal patterns with a peak at around 1000 m. Vegetation recorded at this elevation is represented by beech forests, European hop-hornbeam forests, and mesophilous mixed forests dominated by Italian maple (*Acer opalum* Mill.) [72]. Since litter layers, high porosities associated with soil fauna activities, root proliferation and depth, and many macropores enhance infiltration and percolation rates in forest soils [107], the presence of beech forests might facilitate species that prefer high moisture levels at this elevation, an issue that deserves more investigation. When the overall gradient was divided into two subgradients, we found that the CWM for moisture decreased with an increasing elevation in the first subgradient and decreased in the second one. This is reflected by species distributions outlined by the multi-level analyses, which showed that species associated with humid places predominate at middle elevations, being progressively replaced by species adapted to drier conditions at lower and higher elevations.

The same patterns were observed for the EIVs for nutrients: the CWM for nutrients decreased with an increasing elevation in the first subgradient and decreased in the second one. Multi-level analyses showed how species associated with rich soils predominate at middle elevations, being progressively replaced by species adapted to the scarcity of nutrients at lower and higher elevations. Forest soils are generally characterized by deeply rooted trees, well-developed ‘litter layers’ (O horizons), and the recycling of organic matter and nutrients, including wood [107,108]. Thus, forest soils are rich in nutrients, and this can explain the preponderance of species that prefer high concentrations of nutrients at mid-elevations, which are occupied by forest vegetation.

CWM regressions, fourth-corner analyses, and multi-level analyses indicate that the values of EIVs for continentality do not vary with elevation in any obvious way. This is consistent with our hypothesis of a lack of relationship. We can expect that continentality preferences may vary distinctly with latitude and longitude, as a function of distance from the sea, more than with elevation, at least in short gradients. Thus, it is not surprising that this aspect of ecological preferences is of scarce relevance for our elevational gradient, and confirms that, in general, continentality values vary without meaningful patterns [69,70,71].

As regards the EIVs for reaction (soil pH), contrary to our expectation, we did not find an increase of reaction values with elevation. This suggests that local conditions (namely podzolization and humus forms) that do not vary systematically along the gradient are possibly more important in determining soil pH than elevation [109,110]. In addition, the Ellenberg values for reaction seem to not adequately reflect soil pH, especially for neutral and alkaline soils [39,111,112,113], which may also explain the lack of relationships in multi-level analyses. Interestingly, most of the communities investigated in this study have CWM reaction values between 6 and 7.5, which indicates a prevalence of species associated with slightly basic soils, which is consistent with the prevalence of limestone in the study area. However, there are two sites in which the communities are dominated by species with preferences for relatively acidic soils. These two sites show vegetation types that belong to a phytosociological class (*Nardetea strictae*) typical of places with decalcified, deep, acidic soils [72,76].

Finally, we would stress that our CWM values for the EIVs at the highest elevation were very close to those presented for some other Apennine sites above 2000 m [114], which suggests that our patterns are of general value.

## 5. Conclusions

Our study is the first one to examine how EIVs at the community level vary along an elevational gradient. Plant species do not respond directly to elevation, but rather to changes in abiotic variables regulated by elevation. The use of EIVs allowed us to depict how elevation filters plant species composition and abundance according to their preferences for various abiotic factors. We found that, as expected, temperature preferences showed a distinctly inverse relationship with elevation because temperature decreases with increasing elevation. In contrast to our expectation of a positive monotonic decrease of the sciophilous species, we found that they predominate at middle elevations, because of the presence of shady habitats provided by dense forest cover. Contrary to our expectations, preferences for both soil moisture and nutrients did not correlate linearly with elevation, but showed unimodal patterns, peaking in the middle of the gradient, probably because of the favorable conditions provided by the beech forest soils. EIVs of continentality and reaction (pH) do not vary with elevation in any clear way since these environmental characteristics are probably highly variable locally, a result expected for continentality but not for pH, for which we postulated a positive relationship. These findings indicate that elevation filters plant species according to their environmental preferences in complex, non-obvious ways.

## Figures and Tables

**Table 1:** Description of the investigated communities. For each relevé, the vegetation type is briefly indicated and its syntaxonomic classification at the level of alliance is given, as established by Pirone [72], with nomenclature and higher classification updated according to Prodromo della vegetazione d’Italia [76].

Relevé	Elevation (m)	Description	Alliance	Order/Suborder	Class
1	620	Garrigue	*Cytiso spinescentis-Satureion montanae*	*Cisto cretici-Ericetalia manipuliflorae*	*Cisto cretici-Micromerietea julianae*
2	630	Garrigue	*Cytiso spinescentis-Satureion montanae*	*Cisto cretici-Ericetalia manipuliflorae*	*Cisto cretici-Micromerietea julianae*
3	700	Garrigue	*Cytiso spinescentis-Satureion montanae*	*Cisto cretici-Ericetalia manipuliflorae*	*Cisto cretici-Micromerietea julianae*
4	800	Xerophilous, steppic, and secondary grassland	*Phleo ambigui-Bromion erecti*	*Phleo ambigui-Brometalia erecti*	*Festuco valesiacae-Brometea erecti*
5	980	Hornbeam forest	*Carpinion orientalis*	*Quercetalia pubescenti-petraeae*	*Querco roboris-Fagetea sylvaticae*
6	1110	Mixed mesophilous forest	*Tilio platyphylli-Acerion pseudoplatani*	*Fagetalia sylvaticae*	*Querco roboris-Fagetea sylvaticae*
7	1120	Beech forest	*Geranio versicoloris-Fagion sylvaticae*	*Fagetalia sylvaticae*	*Querco roboris-Fagetea sylvaticae*
8	1630	Xerophilous, steppic, and secondary grassland	*Phleo ambigui-Bromion erecti*	*Phleo ambigui-Brometalia erecti*	*Festuco valesiacae-Brometea erecti*
9	1760	Meso-hygrophilous grassland	*Ranunculo pollinensis-Nardion strictae*	*Nardetalia strictae*	*Nardetea strictae*
10	1830	Mesophilous, acidophilous, and secondary grassland (pasture)	*Ranunculo pollinensis-Nardion strictae*	*Nardetalia strictae*	*Nardetea strictae*
11	1840	Mesophilous and sub-acidophilous grassland	*Ranunculo pollinensis-Nardion strictae*	*Nardetalia strictae*	*Nardetea strictae*
12	1840	Mesophilous, neutral-subacidophilous, and pioneer grassland	*Ranunculo pollinensis-Nardion strictae*	*Nardetalia strictae*	*Nardetea strictae*
13	1850	Mesophilous, neutral-subacidophilous, and pioneer grassland	*Ranunculo pollinensis-Nardion strictae*	*Nardetalia strictae*	*Nardetea strictae*
14	1930	Scree	*Linario-Festucion dimorphae*	*Thlaspietalia stylosi*	*Thlaspietea rotundifolii*
15	2000	Xerophilous, basophilous, pioneer, and enduring grassland	*Seslerion apenninae*	*Seslerienalia apenninae*	*Festuco-Seslerietea*
16	2000	Mesophilous and sub-acidophilous grassland	*Ranunculo pollinensis-Nardion strictae*	*Nardetalia strictae*	*Nardetea strictae*
16	2000	Mesophilous and sub-acidophilous grassland	*Ranunculo pollinensis-Nardion strictae*	*Nardetalia strictae*	*Nardetea strictae*

## Data Availability

All data are given in Tables S1–S5.

## Supplementary Materials

The following supporting information can be downloaded at: https://www.mdpi.com/article/10.3390/biology12020161/s1, Figures S1–S6: relationship between Ellenberg indicator values and elevation excluding trees; Table S1: species abundance (percent cover) with trees; Table S2: EIVs with trees; Table S3: elevation of sites; Table S4: species abundance (percent cover) without trees; Table S5: EIVs without trees; Table S6: results of CWM regression, fourth-corner analysis, and multi-level analysis for temperature with trees; Table S7: results of CWM regression, fourth-corner analysis, and multi-level analysis for light with trees; Table S8: results of CWM regression, fourth-corner analysis, and multi-level analysis for continentality with trees; Table S9: results of CWM regression, fourth-corner analysis, and multi-level analysis for moisture with trees; Table S10: results of CWM regression, fourth-corner analysis, and multi-level analysis for reaction (pH) with trees; Table S11: results of CWM regression, fourth-corner analysis, and multi-level analysis for nutrients with trees; Table S12: results of CWM regression, fourth-corner analysis, and multi-level analysis for temperature without trees; Table S13: results of CWM regression, fourth-corner analysis, and multi-level analysis for light without trees; Table S14: results of CWM regression, fourth-corner analysis, and multi-level analysis for continentality without trees; Table S15: results of CWM regression, fourth-corner analysis, and multi-level analysis for moisture without trees; Table S16: results of CWM regression, fourth-corner analysis, and multi-level analysis for reaction (pH) without trees; and Table S17: results of CWM regression, fourth-corner analysis, and multi-level analysis for nutrients without trees.

## References

[1] Smith T.M., Smith R.L. (2014). Elements of Ecology.

[2] Keddy P.A. (2017). Plant Ecology. Origins, Processes, Consequences.

[3] Schulze E.D., Beck E., Buchmann N., Clemens S., Müller-Hohenstein K., Scherer-Lorenzen M. (2019). Plant Ecology.

[4] ter Braak C.J.F., Šmilauer P. (2003). Multivariate Analysis of Ecological Data Using CANOCO.

[5] Cristóbal E., Ayuso S.V., Justel A., Toro M. (2014). Robust optima and tolerance ranges of biological indicators: A new method to identify sentinels of global warming. Ecol. Res..

[6] Keddy P.A., Laughlin D.C. (2022). A Framework for Community Ecology: Species Pools, Filters, and Traits.

[7] Giller P.S. (1984). Community Structure and the Niche.

[8] Putman R.J. (1994). Community Ecology.

[9] Prinzing A., Durka W., Klotz S., Brandl R. (2002). Geographic variability of ecological niches of plant species: Are competition and stress relevant?. Ecography.

[10] Bruno J.F., Stachowicz J.J., Mark D., Bertness M.D. (2003). Inclusion of facilitation into ecological theory. Trends Ecol. Evol..

[11] Callaway R.M. (2007). Positive Interactions and Interdependence in Plant Communities.

[12] Brooker R.W., Maestre F.T., Callaway R.M., Lortie C.L., Cavieres L.A., Kunstler G., Liancourt P., Tielbörger K., Travis J.M.J., Anthelme F. (2008). Facilitation in plant communities: The past, the present, and the future. J. Ecol..

[13] Smart S.M., Andrew Scott W., Whitaker J., Hill M.O., Roy D.B., Nigel Critchley C., Marini L., Evans C., Emmett B.A., Rowe C. (2010). Empirical realized niche models for British higher and lower plants—Development and preliminary testing. J. Veg. Sci..

[14] Szymura T.H., Szymura M., Macioł A. (2014). Bioindication with Ellenberg’s indicator values: A comparison with measured parameters in Central European oak forests. Ecol. Indic..

[15] Tölgyesi C., Bátori Z., Erdős L. (2014). Using statistical tests on relative ecological indicator values to compare vegetation units—Different approaches and weighting methods. Ecol. Indic..

[16] Ellenberg H. (1952). Landwirtschaftliche Pflanzensoziologie II. Wiesen und Weiden und ihre Standörtliche Bewertung.

[17] Ellenberg H. (1974). Zeigerwerte der Gefäßpflanzen Mitteleuropas.

[18] Ellenberg H. (1979). Zeigerwerte der Gefäßpflanzen Mitteleuropas.

[19] Ellenberg H., Weber H.E., Düll R., Wirth V., Werner W., Paulissen D. (1991). Zeigerwerte von Pflanzen in Mitteleuropa.

[20] Ellenberg H., Weber H.E., Düll R., Wirth V., Werner W., Paulissen D. (1992). Zeigerwerte von Pflanzen in Mitteleuropa. 2. Verbesserte und Erweiterte Auflage.

[21] Ellenberg H., Weber H.E., Düll R., Wirth V., Werner W., Paulissen D. (2001). Zeigerwerte von Pflanzen in Mitteleuropa. 3. Durchgesehene Auflage.

[22] Zarzycki K., Trzcińska-Tacik H., Różański W., Szeląg Z., Wołek J., Korzeniak U. (2002). Ecological Indicator Values of Vascular Plants of Poland. Ekologiczne Liczby Wskaźnikowe Roślin Naczyniowych Polski.

[23] Borhidi A. (1995). Social behaviour types, the naturalness and relative ecological indicator values of the higher plants in theungariann flora. Acta Bot. Hungar..

[24] Pignatti S., Bianco P.M., Fanelli G., Guarino R., Petersen L., Tescarollo P., Walter G.R., Burga C.A., Edwards P.J. (2001). Reliability and Effectiveness of Ellenberg’s Indices in Checking Flora and Vegetation Changes Induced by Climatic Variations, In Fingerprints of Climate Changes: Adapted Behaviour and Shifting Species Ranges.

[25] Pignatti S., Menegoni P., Pietrosanti S. (2005). Valori di biondicazione delle piante vascolari della flora d’Italia. Braun-Blanquetia.

[26] Böhling N., Greuter W., Raus T. (2002). Indicator values for vascular plants in the Southern Aegean (Greece). Braun-Blanquetia.

[27] Gégout J.C., Krizova E. (2003). Comparison of indicator values of forest understory plant species in Western Carpathians (Slovakia) and Vosges Mountains (France). For. Ecol. Manag..

[28] Hill M.O., Mountford J.O., Roy D.B., Bunce R.G.H. (1999). Ellenberg’s Indicator Values for British Plants.

[29] Hill M.O., Roy D.B., Owen Mountford J., Bunce R.G.H. (2000). Extending Ellenber’s Indicator Values to a New Area: An Algorithmic Approach. J. Appl. Ecol..

[30] Lawesson J.E., Fosaa A.M., Olsen E. (2003). Calibration of Ellenberg indicator values for Faroe islands. Appl. Veg. Sci..

[31] Kosić I.V., Juračak J., Łuczaj Ł. (2017). Using Ellenberg-Pignatti values to estimate habitat preferences of wild food and medicinal plants: An example from northeastern Istria (Croatia). J. Ethnobiol. Ethnomedicine.

[32] Hedwall P.-O., Brunet J., Diekmann M. (2019). With Ellenberg indicator values towards the north: Does the indicative power decrease with distance from Central Europe?. J. Biogeogr..

[33] Chytrý M., Tichý L., Dřevojan P., Sádlo J., Zelený D. (2018). Ellenberg-type indicator values for the Czech flora. Preslia.

[34] Hill M.O., Carey P.D. (1997). Prediction of yield in the Rothamsted Park Grass Experiment by Ellenberg indicator values. J. Veg. Sci..

[35] Van Dobben H.F., ter Braak C.J.F., Dirkse G.M. (1999). Undergrowth as a biomonitor for deposition of nitrogen and acidity in pine forest. For. Ecol. Manag..

[36] Bergès L., Gégout J.C., Franc A. (2006). Can understory vegetation accurately predict site index? A comparative study using floristic and abiotic indices in sessile oak (*Quercus petraea* Liebl.) stands in northern France. Ann. For. Sci..

[37] Wagner M., Kahmen A., Schlumprecht H., Audorff V., Perner J., Buchmann N., Weisser W.W. (2007). Prediction of herbage yield in grassland: How well do Ellenberg N-values perform?. Appl. Veg. Sci..

[38] Axmanová I., Tichý L., Fajmonová Z., Hájková P., Hettenbergerová E., Li C.F., Merunková K., Najzchlebová M., Otýpková Z., Vymazalová M. (2012). Estimation of herbaceous biomass from species composition and cover. Appl. Veg. Sci..

[39] Häring T., Reger B., Ewald J., Hothorn T., Schröder B. (2012). Predicting Ellenberg’s soil moisture indicator value in the Bavarian Alps using additive georegression. Appl. Veg. Sci..

[40] Hawkes J.C., Pyatt D.G., White I.M.S. (1997). Using Ellenberg Indicator Values to assess soil quality in British forests from ground vegetation: A pilot study. J. App. Ecol..

[41] Ertsen A.C.D., Alkemade J.R.M., Wassen M.J. (1998). Calibrating Ellenberg indicator values for moisture, acidity, nutrient availability and salinity in the Netherlands. Plant Ecol..

[42] Diekmann M. (2003). Species indicator values as an important tool in applied plant ecology—A review. Basic Appl. Ecol..

[43] Wamelink G.W., Goedhart P.W., Van Dobben H.F., Berendse F. (2005). Plant species as predictors of soil pH: Replacing expert judgement with measurements. J. Veg. Sci..

[44] Bartelheimer M., Poschlod P. (2016). Functional characterizations of Ellenberg indicator values—A review on ecophysiological determinants. Funct. Ecol..

[45] Persson S. (1981). Ecological Indicator Values as an Aid in the Interpretation of Ordination Diagrams. J. Ecol..

[46] Major J., Rejmanek M. (1992). *Amelanchier alnifolia* vegetation in eastern Idaho, USA and its environmental relationships. Vegetatio.

[47] Thompson K., Hodgson J.G., Grime J.P., Rorison I.H., Band S.R., Spencer R.E. (1993). Ellenberg numbers revisited. Phytocoenologia.

[48] Marcenò C., Guarino R.A. (2015). A test on Ellenberg indicator values in the Mediterranean evergreen woods (*Quercetea ilicis*). Rend. Fis. Acc. Lincei.

[49] Schaffers A.P., Sýkora K.V. (2000). Reliability of Ellenberg indicator values for moisture, nitrogen and soil reaction: A comparison with field measurements. J. Veg. Sci..

[50] Wamelink G.W.W., Joosten V., van Dobben H.H., Berendse F. (2002). Validity of Ellenberg indicator values judged from physico-chemical field measurements. J. Veg. Sci..

[51] Chytrý M., Tichý L., Roleček J. (2002). Local and regional patterns of species richness in Central European vegetation types along the pH/calcium gradient. Folia Geobot..

[52] Sørensen M.M., Tybirk K. (2000). Vegetation analysis along a successional gradient from heath to oak forest. Nord. J. Bot..

[53] Lososová Z., Chytrý M., Cimalová S., Kropáč Z., Otýpková Z., Pyšek P., Tichý L. (2004). Weed vegetation of arable land in Central Europe: Gradients of diversity and species composition. J. Veg. Sci..

[54] Fraaije R.G.A., ter Braak C.J.F., Verduyn B., Breeman L.B.S., Verhoeven J.T.A., Soons M.B. (2015). Early plant recruitment stages set the template for the development of vegetation patterns along a hydrological gradient. Funct. Ecol..

[55] Kutbay H., Surmen B. (2022). Ellenberg ecological indicator values, tolerance values, species niche models for soil nutrient availability, salinity, and pH in coastal dune vegetation along a landward gradient (Euxine, Turkey). Turk. J. Bot..

[56] Körner C. (2003). Alpine Plant Life: Functional Plant Ecology of High Mountain Ecosystems.

[57] Sundqvist M.K., Sanders N.J., Wardle D.A. (2013). Community and ecosystem responses to elevational gradients: Processes, mechanisms, and insights for global change. Annu. Rev. Ecol. Evol. Syst..

[58] Fattorini S., Di Biase L., Chiarucci A. (2019). Recognizing and interpreting vegetational belts: New wine in the old bottles of a von Humboldt’s legacy. J. Biogeogr..

[59] Fattorini S., Mantoni C., Di Biase L., Pace L., Leal Filho W., Azul A., Brandli L., Özuyar P., Wall T. (2020). Mountain biodiversity and sustainable development. Encyclopedia of the UN Sustainable Development Goals. Life on Land.

[60] Fattorini S., Mantoni C., Di Biase L., Strona G., Pace L., Biondi M. (2020). Elevational patterns of generic diversity in the tenebrionid beetles (Coleoptera Tenebrionidae) of Latium (Central Italy). Diversity.

[61] Moradi H., Fattorini S., Oldeland J. (2020). Influence of elevation on the species–area relationship. J. Biogeogr..

[62] Bruelheide H., Dengler J., Purschke O., Lenoir J., Jiménez-Alfaro B., Hennekens S.M., Botta-Dukát Z., Chytrý M., Field R., Jansen F. (2018). Global trait-environment relationships of plant communities. Nat. Ecol. Evol..

[63] Boonman C.C.F., Santini L., Robroek B.J.M., Hoeks S., Kelderman S., Dengler J., Bergamini A., Biurrun I., Carranza M.L., Cerabolini B.E.L. (2021). Plant functional and taxonomic diversity in European grasslands along climatic gradients. J. Veg. Sci..

[64] Hillman P. (1985). The Basics of Biogeography.

[65] Jobbagy E.G., Jackson R.B. (2000). The vertical distribution of soil organic carbon and its relation to climate and vegetation. Ecol. Appl..

[66] Smith J.L., Halvorson J.J., Bolton H. (2002). Soil properties and microbial activity across a 500 m elevation gradient in a semi-arid environment. Soil Biol. Biochem..

[67] Northcott M.L., Gooseff M.N., Barrett J.E., Zeglin L.H., Takacs-Vesbach C.D., Humphrey J. (2009). Hydrologic characteristics of lake and stream-side riparian margins in the McMurdo Dry Valleys, Antarctica. Hydrol. Process.

[68] Jeyakumar S.P., Dash B., Singh A.K., Suyal D.C., Soni R., Goel R., Soni R., Suyal D.C. (2020). Nutrient cycling at higher altitudes. Microbiological Advancements for Higher Altitude Agro-Ecosystems & Sustainability.

[69] Berg C., Welk E., Jäger E.J. (2017). Revising Ellenberg’s indicator values for continentality based on global vascular plant species distribution. Appl. Veg. Sci..

[70] Fischer H.S., Michler B., Ewald J. (2014). Environmental, spatial and structural components in the composition of mountain forest in the Bavarian alps. Folia Geobot..

[71] Koch M., Jurasinski G. (2014). Four decades of vegetation development in a percolation mire complex following intensive drainage and abandonment. Plant Ecol. Diver..

[72] Pirone G., Burri E. (1998). Aspetti della vegetazione della riserva naturale guidata monte Genzana e Alto Gizio. Aree protette in Abruzzo. Contributi alla Conoscenza Naturalistica ed Ambientale.

[73] Piano di Gestione del Sito di Interesse Comunitario IT 7110100 Monte Genzana. http://www.riservagenzana.it/pdf/Pdg_Monte_Genzana.pdf..

[74] Di Biase L., Pace L., Mantoni C., Fattorini S. (2021). Variations in plant richness, biogeographical composition, and life forms along an elevational gradient in a Mediterranean mountain. Plants.

[75] Pignatti S., Guarino R., La Rosa M. (2017–2019). Flora dʹItalia.

[76] Prodromo della Vegetazione d’Italia. https://www.prodromo-vegetazione-italia.org/introduzione.

[77] Braun-Blanquet J. (1964). Pflanzensoziologie. Grundzüge der Vegetationskunde.

[78] Hennekens S.M., Schaminée J.H.J. (2001). Turboveg, a Comprehensive Data Base Management System for Vegetation Data. J. Veg. Sci..

[79] Pätsch R., Jašková A., Chytrý M., Kucherov I.B., Schaminée J.H.J., Bergmeier E., Janssen J.A.M. (2019). Making them visible and usable—Vegetation-plot observations from Fennoscandia based on historical species-quantity scales. Appl. Veg. Sci..

[80] Tichý L., Hennekens S.M., Novák P., Rodwell J.S., Schaminée J.H.J., Chytrý M. (2020). Optimal transformation of species cover for vegetation classification. Appl. Veg. Sci..

[81] Guarino R., Domina G., Pignatti S. (2012). Ellenberg’s Indicator values for the Flora of Italy—First update: Pteridophyta, Gymnospermae and Monocotyledoneae. Flora Mediterr..

[82] Garnier E., Cortez J., Neill C., Toussaint J.-P., Billès G., Navas M.-L., Roumet C., Debussche M., Laurent G., Blanchard A. (2004). Plant functional markers capture ecosystem properties during secondary succession. Ecology.

[83] Ricotta C., Moretti M. (2011). CWM and Rao’s quadratic diversity: A unified framework for functional ecology. Oecologia.

[84] Di Biase L., Fattorini S., Cutini M., Bricca A. (2021). The role of inter-and intraspecific variations in grassland plant functional traits along an elevational gradient in a Mediterranean mountain area. Plants.

[85] Chapman H., Cordeiro N.J., Dutton P., Wenny D., Kitamura S., Kaplin B., Melo F.P., Lawes M.J. (2016). Seed-dispersal ecology of tropical montane forests. J. Trop. Ecol..

[86] Funk J.L., Larson J.E., Ames G.M., Butterfield B.J., Cavender-Bares J., Firn J., Laughlin D.C., Sutton-Grier A.E., Williams L., Wright J. (2017). Revisiting the holy grail: Using plant functional traits to understand ecological processes. Biol. Rev..

[87] ter Braak C.J.F., Peres-Neto P.R., Dray S. (2018). Simple parametric tests for trait-environment association. J. Veg. Sci..

[88] Hanif M.A., Yu Q., Rao X., Shen W. (2019). Disentangling the Contributions of Plant Taxonomic and Functional Diversities in Shaping Aboveground Biomass of a Restored Forest Landscape in Southern China. Plants.

[89] Rolhauser A.G., Waller D.M., Tucker C.M. (2021). Complex trait–environment relationships underlie the structure of forest plant communities. J. Ecol..

[90] Cheng X., Ping T., Li Z., Tian W., Hairong H., Epstein H.E. (2022). Effects of environmental factors on plant functional traits across different plant life forms in a temperate forest ecosystem. New For..

[91] ter Braak C.J.F., Peres-Neto P.R., Dray S. (2017). A critical issue in model-based inference for studying trait-based community assembly and a solution. PeerJ.

[92] Miller J.E.D., Damschen E.I., Ives A.R. (2019). Functional traits and community composition: A comparison among community-weighted means, weighted correlations, and multilevel models. Methods Ecol. Evol..

[93] Dray S., Legendre P. (2008). Testing the species traits–environment relationships: The fourth-corner problem revisited. Ecology.

[94] Peres-Neto P.R., Dray S., ter Braak C.J.F. (2017). Linking trait variation to the environment: Critical issues with community-weighted mean correlation resolved by the fourth-corner approach. Ecography.

[95] Brown A.M., Warton D.I., Andrew N.R., Binns M., Cassis G., Gibb H. (2014). The fourth-corner solution—Using predictive models to understand how species traits interact with the environment. Methods Ecol. Evol..

[96] Laughlin D.C., Gremer J.R., Adler P.B., Mitchell R.M., Moore M.M. (2020). The Net Effect of Functional Traits on Fitness. Trends Ecol. Evol..

[97] Gelman A., Hill J. (2006). Data Analysis Using Regression and Multilevel/Hierarchical Models.

[98] Laughlin D.C., Strahan R.T., Adler P.B., Moore M.M. (2018). Survival rates indicate that correlations between community weighted mean traits and environments can be unreliable estimates of the adaptive value of traits. Ecol. Lett..

[99] R Core Team (2020). R: A Language and Environment for Statistical Computing.

[100] Laughlin D. R Code and Data for “A Framework for Community Ecology: Species Pools, Traits, and Filter” by Paul Keddy and Daniel Laughlin. https://github.com/danielLaughlin/CommunityEcology/blob/master/communityEcology.R.

[101] Laliberté E., Legendre P., Shipley B. (2014). FD: Measuring Functional Diversity from Multiple Traits, and Other Tools for Functional Ecology. R Package Version 1.0-12.1. https://cran.r-project.org/web/packages/FD/index.html.

[102] Oksanen J., Simpson G.L., Blanchet F.G., Kindt R., Legendre P., Minchin P.R., O’Hara R.B., Solymos P., Stevens M.H.H., Szoecs E. ‘Vegan’: Community Ecology Package. R Package Version 2.6–4. https://CRAN.R-project.org/package=vegan.

[103] Dray S., Dufour A.-B., Thioulouse J. ade4: Analysis of Ecological Data: Exploratory and Euclidean Methods in Environmental Sciences.R Package Version 1.7–20. https://cran.r-project.org/web/packages/ade4/index.html.

[104] Bates D., Maechler M., Bolker B., Walker S. (2021). lme4: Linear Mixed-Effects Models Using Eigen and S4. R Package Version 1.1-31. https://cran.r-project.org/web/packages/lme4/.

[105] Lüdecke D., Makowski D., Ben-Shacar M.S., Patil I., Waggoner P., Wiernik B.M. performance: Assessment of Regression Models Performance. R Package Version 0.10.1. https://cran.r-project.org/web/packages/performance/index.html.

[106] Chelli S., Marignani M., Barni E., Petraglia A., Puglielli G., Wellstein C., Acosta A.T.R., Bolpagni R., Bragazza L., Campetella G. (2018). Plant-environment interactions through a functional traits perspective: A review of Italian studies. Plant Biosyst..

[107] Boyle J.R., Hillel D. (2005). Forest Soils. Encyclopedia of Soils in the Environment.

[108] Ranger J., Berthelin J., Valentin C., Munch J.C. (2018). Forest Soils: Characteristics and Sustainability. Soils as a Key Component of the Critical Zone 1: Functions and Services.

[109] Bhandari J., Zhang Y. (2019). Effect of altitude and soil properties on biomass and plant richness in the grasslands of Tibet, China, and Manang District, Nepal. Ecosphere.

[110] Badía D., Ruiz A., Girona A., Martí C., Casanova J., Ibarra P., Zufiaurre R. (2016). The influence of elevation on soil properties and forest litter in the Siliceous Moncayo Massif, SW Europe. J. Mt. Sci..

[111] Lawesson J.E. (2003). pH optima for Danish forest species compared with Ellenberg reaction values. Folia Geobot..

[112] Balkovič J., Kollár J., Šimonovič V. (2012). Experience with using Ellenberg’s R indicator values in Slovakia: Oligotrophic and mesotrophic submontane broad-leaved forests. Biologia.

[113] Carpenter W., Goodenough A. (2014). How robust are community-based plant bioindicators? Empirical testing of the relationship between Ellenberg values and direct environmental measures in woodland communities. Comm. Ecol..

[114] Bricca A., Carranza M.L., Varricchione M., Cutini M., Stanisci A. (2021). Exploring Plant Functional Diversity and Redundancy of Mediterranean High-Mountain Habitats in the Apennines. Diversity.

