# Supplementary material for: Ellenberg Indicator Values Disclose Complex Environmental Filtering Processes in Plant Communities along an Elevational Gradient
Source: Biology (Basel). 2023 Jan 19;12(2):161. doi: 10.3390/biology12020161 (PMC9953212; doi:10.3390/biology12020161)
Supplement: Supplementary file 1 [file biology-12-00161-s001.zip › Figures S1-S6.pdf]

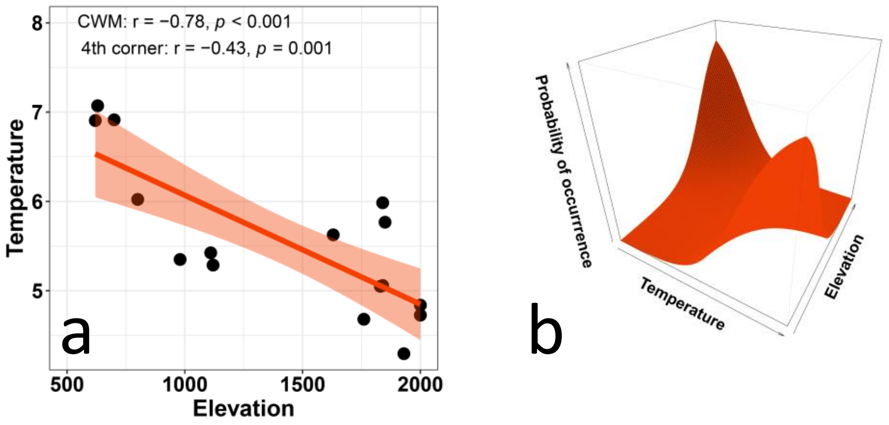

Figure S1. Relationship between Ellenberg indicator values for temperature and elevation in plant communities along an elevational gradient in Central Italy. The left panel (**a**) presents the CWM regression model and statistical corrections based on the fourth corner analysis. The right panel (**b**) presents the results of the multi-level model (trait x environment interaction $p < 0.001$, marginal R^2=0.06).

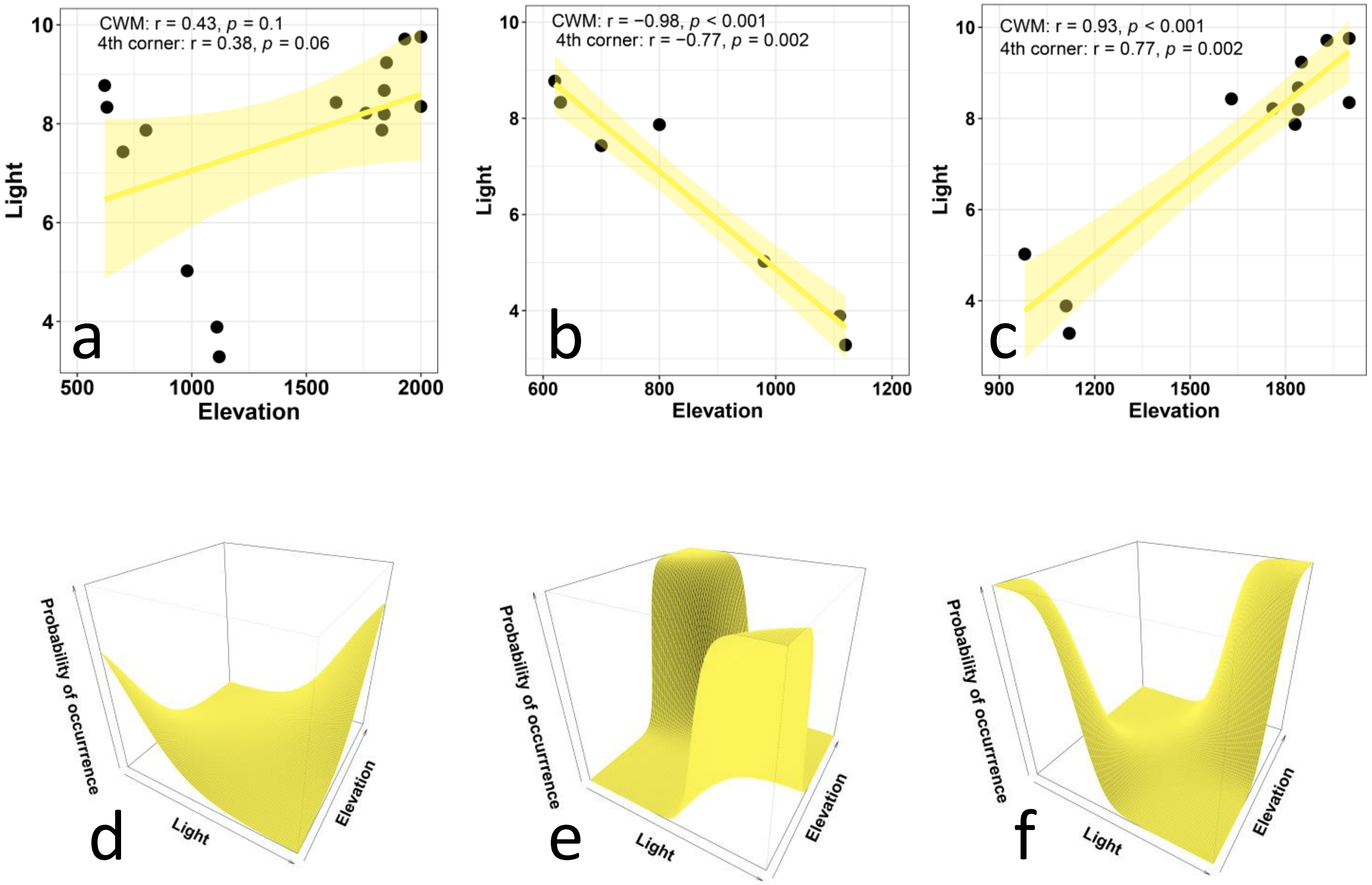

Figure S2. Relationship between Ellenberg indicator values for light and elevation in plant communities along an elevational gradient in Central Italy. The upper panels (**a-d**) present CWM regression models and their statistical corrections using fourth corner analysis for the entire gradient (**a**), for the lower subgradient (**b**), and for the upper subgradient (**c**). The lower panels (**d-f**) illustrate the results of multi-level models for the entire gradient (**d**, trait x environment interaction $p < 0.001$, marginal $R^2 = 0.03$), for the lower subgradient (**e**, trait x environment interaction $p < 0.001$, marginal $R^2 = 0.31$), and for the upper subgradient (**f**, trait x environment interaction $p < 0.001$, marginal $R^2 = 0.25$).

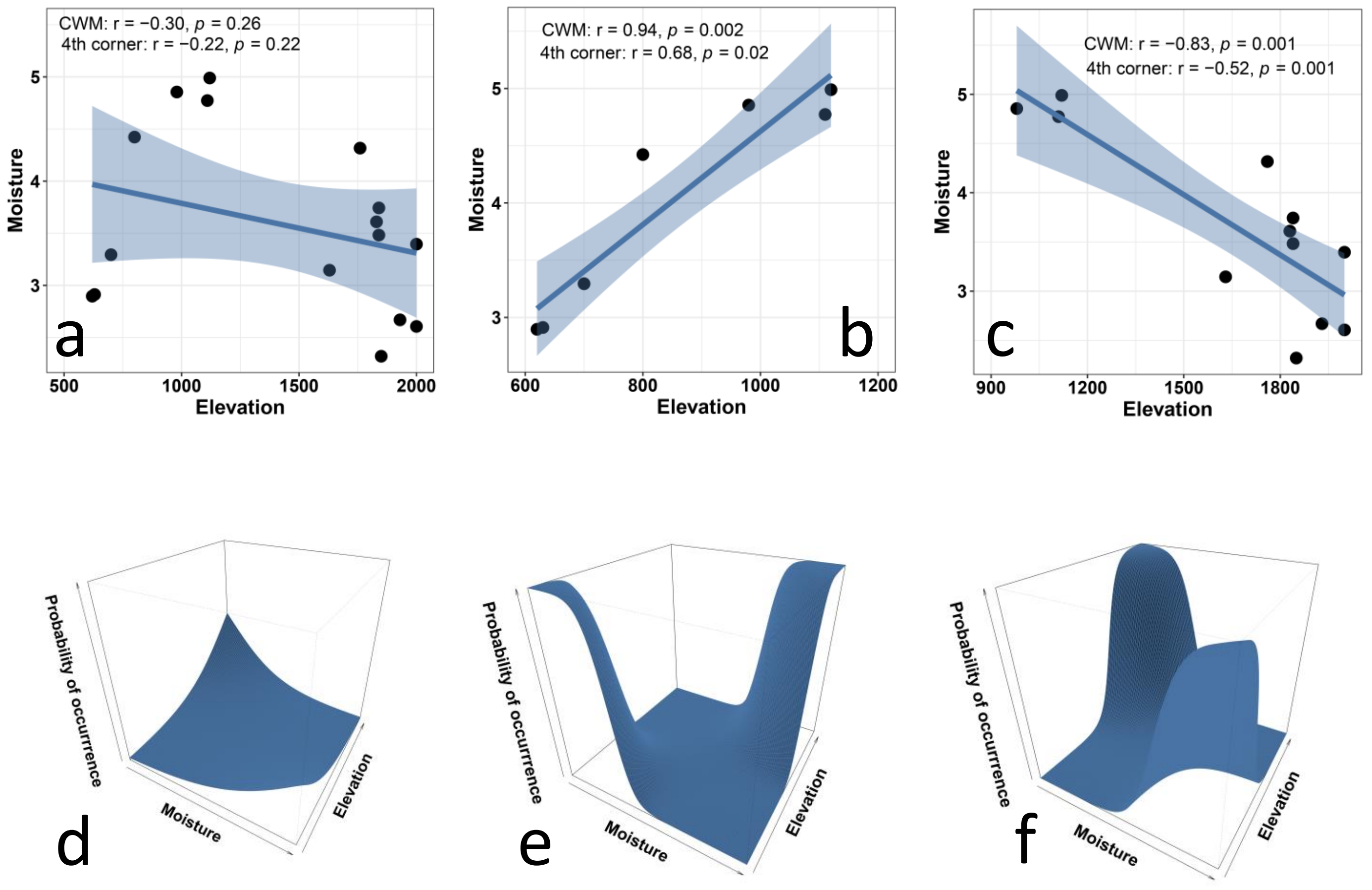

Figure S3. Relationship between Ellenberg indicator values for moisture and elevation in plant communities along an elevational gradient in Central Italy. The upper panels (**a-d**) present CWM regression models and their statistical corrections using fourth corner analysis for the entire gradient (**a**), for the lower subgradient (**b**), and for the upper subgradient (**c**). The lower panels (**d-f**) illustrate the results of multi-level models for the entire gradient (**d**, trait x environment interaction $p < 0.01$, marginal $R^2 = 0.01$), for the lower subgradient (**e**, trait x environment interaction $p < 0.001$, marginal $R^2 = 0.22$), and for the upper subgradient (**f**, trait x environment interaction $p < 0.001$, marginal $R^2 = 0.14$).

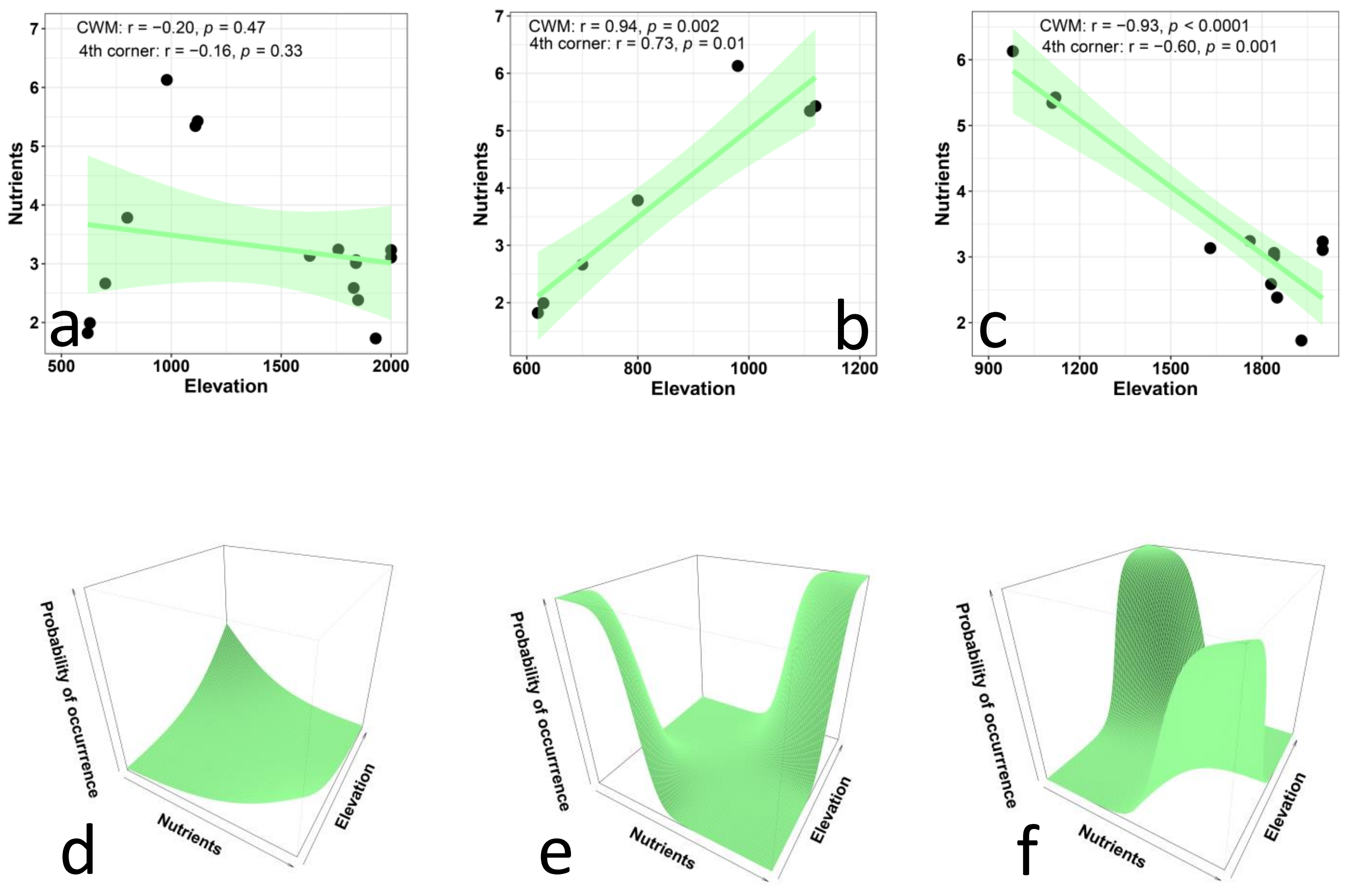

Figure S4. Relationship between Ellenberg indicator values for nutrients and elevation in plant communities along an elevational gradient in Central Italy. The upper panels (**a-d**) present CWM regression models and their statistical corrections using fourth corner analysis for the entire gradient (**a**), for the lower subgradient (b), and for the upper subgradient (**c**). The lower panels (**d-f**) illustrate the results of multi-level models for the entire gradient (**d**, trait x environment interaction $p < 0.01$, marginal $R^2 = 0.01$), for the lower subgradient (**e**, trait x environment interaction $p < 0.001$, marginal $R^2 = 0.23$), and for the upper subgradient (**f**, trait x environment interaction $p < 0.001$, marginal $R^2 = 0.15$).

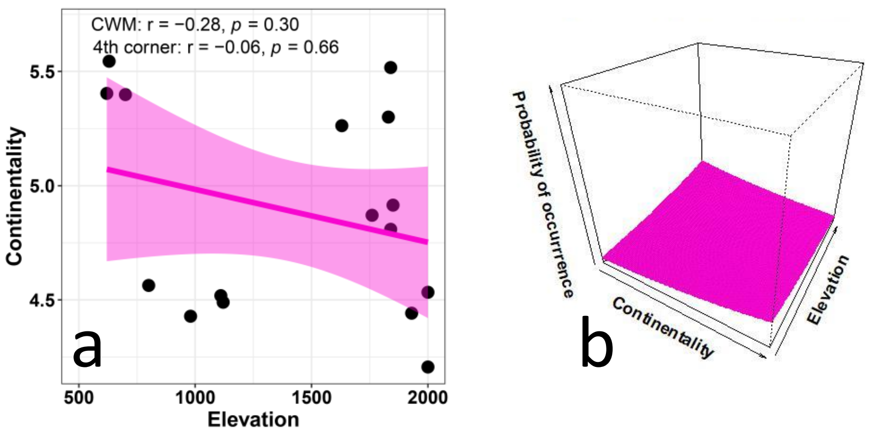

Figure S5. Relationship between Ellenberg indicator values for continentality and elevation in plant communities along an elevational gradient in Central Italy. The left panel (**a**) presents the CWM regression model and statistical corrections based on the fourth corner analysis. The right panel (**b**) presents the results of the multi-level model (trait x environment interaction $p = 0.32$, marginal $R^2 = 0.002$).

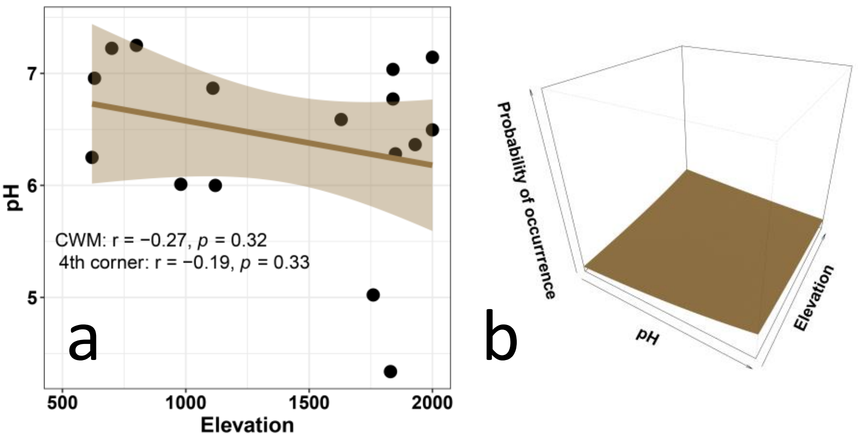

Figure S6. Relationship between Ellenberg indicator values for reaction (pH) and elevation in plant communities along an elevational gradient in Central Italy. The left panel (**a**) presents the CWM regression model and statistical corrections based on the fourth corner analysis. The right panel (**b**) presents the results of the multi-level model (trait x environment interaction $p = 0.46$, marginal $R^2 = 0.003$).
